# Evaluation of dietary supplement, functional food and herbal medicine use by dietitians during the COVID-19 pandemic

**DOI:** 10.1017/S1368980020005297

**Published:** 2020-12-28

**Authors:** Hulya Kamarli Altun, Merve Seyda Karacil Ermumcu, Nilgun Seremet Kurklu

**Affiliations:** Department of Nutrition and Dietetics, Faculty of Health Sciences, Akdeniz University, Antalya, Turkey

**Keywords:** COVID-19, Dietary supplements, Dietitians, Functional foods, Herbal medicine

## Abstract

**Objective:**

The current study was conducted to evaluate the dietary habits of the dietitians who had a leading role in this regard during the pandemic and their use of dietary supplements, functional food and herbal medicines.

**Design:**

A cross-sectional study. An online questionnaire was used as a data collection tool to identify the participants’ socio-demographic characteristics, health statuses and dietary habits and their use of dietary supplements, functional foods and herbal medicines.

**Setting:**

Turkey.

**Participants:**

The study population was 550 dietitians.

**Results:**

In the current study, the participants’ average age was 30·6 ± 9·1 years, and most of them (88·2 %) were women. More than half of the participants (88·9 %) thought that adequate and balanced nutrition would positively affect the course of COVID-19. To avoid COVID-19, 94·5 % of the dietitians used dietary supplements, 46·1 % herbal medicines and 34·9 % functional foods during the pandemic. The most commonly used dietary supplement was fish oil (81·9 %), functional food was vegetables and fruits (80·5 %) and the herbal medicine was cinnamon (63·5 %). Women’s consumption of functional foods was approximately twice higher compared with men (95 % Cl: 1·048, 4·165; *P* < 0·05). The findings showed that the longer the dietitians were in their careers, the more functional foods and herbal medicines they used.

**Conclusion:**

During the pandemic, dietitians’ use of foods with protective effects against diseases increased depending on their academic knowledge and experience in nutrition. The findings obtained in the current study suggest that an expert’s opinion should be obtained before using dietary supplements and herbal medicines.

## Graphical abstract

**Figure:**
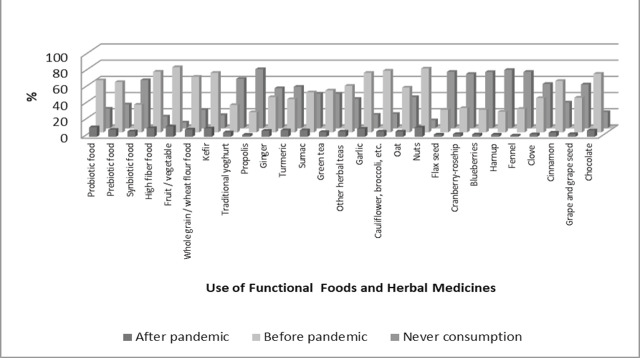

Coronaviruses are a large family of viruses that may cause diseases in animals or humans^(1)^. Novel Coronavirus Disease 2019 (COVID-19), on the other hand, is a disease that arises from the most recently discovered virus from this family, is transmitted by droplets and has a high level of contagiousness. COVID-19 was first reported in Wuhan, China, in December 2019 and caused a pandemic. COVID-19, which took the world under its influence, was declared as pandemic by the WHO on 19 March 2020, and 28·918·900 confirmed cases and 922 252 deaths were reported worldwide (data of 14 September 2020)^(2–4)^. In this pandemic, our country ranked seventh in April, following the USA, Spain, Italy, France, Germany and England concerning the number of cases and eighteenth in September with 291 162 confirmed cases and 7056 deaths^(4)^.

Approximately 80 % of the people infected with the COVID-19 virus experience mild to moderate respiratory distress and recover without the need for any special treatment. However, it has been observed that the disease progresses more severely in the elderly and in individuals with accompanying chronic diseases, such as CVD, diabetes, chronic respiratory disease and cancer. Currently, no vaccine or drug treatment has been developed for the novel coronavirus^(1,5)^. Thus, our natural immune system should be strong enough to fight it.

The immune system is affected primarily by genetic factors, and among environmental factors, by dietary habits. Therefore, in this period, individuals have started to pay attention to adequate and balanced nutrition more than ever to boost their immune systems to avoid the disease or to overcome it with minimal damage. In particular, the consumption of vegetables and fruit groups rich in antioxidants and vitamins and minerals and foods that constitute rich protein sources has increased^(6)^. Also, it is thought that the number of people who use dietary supplements to have an adequate and balanced diet and who use them to improve body resistance may have increased considerably.

A dietary supplement is defined as the usable forms of the amounts corresponding to high doses of vitamins and minerals and refers to supplements to the nutrients in our diet. Dietary supplements are not needed for an adequate and balanced diet^(7)^. Dietary supplements may cause toxic effects when taken in uncontrolled high doses/amounts for a long time. They should only be used if recommended by a physician for a specific disease or for individuals who cannot obtain them adequately in their diet. However, since they are easily accessible in pharmacies, the consumption of vitamin and mineral supplements by individuals without the need for any advice may climb, especially during periods when infectious diseases emerge.

In addition to dietary supplements, it is predicted that the consumption of functional foods and herbal medicines may increase with the pandemic. Functional foods are natural or processed foods that contain known or unknown biologically active compounds that are effective, non-toxic and capable of regulating body functions. Functional foods are not drugs or any dietary supplements but are part of the regular food/nutritional pattern. Carotenoids, *n*-3 fatty acids, isoflavones, flavonoids, isocyanates, phenolic acids, phytooestrogens, polyphenols, soluble dietary fibres, plant stanols and sterols, polyols, probiotics, prebiotics and synbiotic are defined as functional food components. These nutritional components play a significant role in maintaining health and reducing the risk of chronic diseases^(8–12)^. It is stated that functional foods have beneficial effects on respiratory diseases, such as asthma, many viral and parasitic diseases, infectious diseases, inflammatory diseases and psychotic disorders^(9,10)^.

Herbal medicines, on the other hand, are defined as plants (such as leaves, roots and stems) and substances produced from one or more plants^(13)^. In this period, there may be a rise in the consumption of herbal medicines due to the increase in the tendency to produce individual solutions to health problems. However, their use should be controlled since they contain components that have negative effects, as well as positive effects, on health, and they may interact with drugs used in the treatment of some diseases^(7,14,15)^.

In a pandemic, individuals’ tendency towards foods and nutrients that have protective or therapeutic effects may increase. In this period, dietitians have assumed a critical role in nutrition, which has crucial effects on the prevention and treatment of the disease. They have provided consultancy services for individuals on the relevant nutrients, foods and dietary habits during the disease prevention process. However, individual approaches of the dietitians, who are well informed about dietary supplements, functional foods and herbal medicines and are following the current literature on this subject, have not been evaluated during the COVID-19 pandemic. The current study was conducted to evaluate dietitians’ use of dietary supplements, functional foods and herbal medicines.

## Material and methods

This cross-sectional study was conducted between May 2020 and June 2020 to investigate the dietitians’ use of dietary supplements, functional foods and herbal medicines during the pandemic. The population of the study consisted of nearly 18 400 dietitians who graduated from the Department of Nutrition and Dietetics of the universities in Turkey. The sample of the current study was defined as 526 people with a 5 % error and a 98 % CI in the G-power program, based on the calculation of a sample with a known universe. At the end of the current study, 583 dietitians who consented and agreed to participate in the current study were contacted. Thirty-three people with missing data in the questionnaire were excluded from the current study, and 550 people were included in the current study. Due to the pandemic, an online questionnaire was used as a data collection tool, and the questionnaire was delivered to the dietitians using digital means, such as social media, dietitian accounts and e-mail. The questionnaire applied to the participants included questions about their socio-demographic characteristics, health statuses, dietary habits and the frequency of their use of dietary supplements, functional foods and herbal medicines. The frequency of use of fifty-seven functional foods and herbal medicines by the participants before and after the pandemic was investigated. BMI values were calculated based on the body weight and height obtained from the participants’ statements, using the body weight (kg)/height^2^ (m^2^) formula. WHO criteria were used in the classification of BMI.

### Statistical analyses

The data in the current study were analysed using SPSS 22.0 statistical package software. The results were summarised using descriptive statistics, and *χ* ^2^ analysis was used in the comparison of the qualitative data. The effects of some independent variables (gender, age, career year, obesity and disease) on the use of functional foods and herbal medicines were analysed using logistic regression analysis. Hosmer–Lemeshow test was used for model fit. The level of significance was accepted as *P* < 0·05 for all statistical analyses.

## Results

The average age of the individuals participating in the current study was 30·6 ± 9·1 years, and it was observed that the majority of them (88·2 %) were women. The average BMI of the participants was 22·4 ± 4·36 kg/m^2^, and BMI values of approximately three out of every four dietitians were normal. The majority of the participants had a bachelor’s degree and worked in public institutions (60·7 and 46·5 %, respectively). According to the statements of the participants, 82·0 % of them did not have any chronic diseases, and approximately half of those who did had endocrine system diseases (48·5 %). While 39·6 % of the dietitians had vitamin-mineral deficiency diagnosed by a doctor, 87·2 % of them used vitamin-mineral supplements for treatment (Table 1).

**Table 1:** Evaluation of demografic characteristics and health information of dietitians

	*n*	%
Gender		
Female	485	88·2
Male	65	11·8
BMI		
Underweight (< 18·5 kg/m^2^)	52	9·5
Normal (18·5–24·9 kg/m^2^)	403	73·3
Overweight (25·0–29·9 kg/m^2^)	69	12·5
Obese (>30 kg/m^2^)	26	4·7
Level of Education		
Bachelor	334	60·7
Master’s degree	161	29·3
Doctoral (PhD)	55	10·0
Marital status		
Married	225	40·9
Single	325	59·1
Job Type		
Public institutions	256	46·5
Private practice	188	34·2
Retired	11	2·0
Not working	95	17·3
Having chronic disease	99	28·0
Types of chronic disease		
Endocrine diseases	48	48·5
Neurological diseases	12	12·1
Allergic diseases	11	11·1
Respiratory system diseases	8	8·1
Digestive system diseases	11	11·1
Rheumatic diseases	3	3·0
CVDs	6	6·1
Having vitamin-mineral deficiency	218	39·6
Using vitamin-mineral supplements	190	87·2
Smoking	90	16·4
Alcohol use	133	24·7

When the dietitians’ dietary habits during the pandemic were analysed, it was seen that the majority of them had two main meals and two snacks a day (54·2 and 48·0 %, respectively). The majority of the participants (88·9 %) stated that adequate and balanced nutrition would positively affect the course of COVID-19. Approximately three out of four dietitians thought that they had an adequate and balanced diet during the pandemic period, and the number of dietitians who stated that their eating habits changed positively during the pandemic and that of dietitians who observed no change in their diet were close (35·1 and 35·3 %, respectively) (Table 2).

**Table 2:** Dietary habits of dietitians during the pandemic period

	*n*	%
Number of main meals		
1	7	1·3
2	298	54·2
≥ 3	245	44·5
Number of snacks		
1	185	33·7
2	264	48·0
≥ 3	101	13·3
Changing eating habits during pandemic		
Yes, Positively/more healthy	193	35·1
Yes, Negatively/more unhealthy	163	29·6
No	194	35·3
Having adequate and balanced nutrition during the pandemic period		
Yes	406	73·8
No	144	26·2
Adequate and balanced nutrition positively affects the course of COVID-19		
Yes	489	88·9
No	61	11·1

The participants’ use of dietary supplements during the pandemic was questioned, and it was found that the number of dietitians who did not use dietary supplements and that of dietitians who did before the pandemic were close (43·6 and 42·4 %, respectively), and more than half of the dietitians (54·7 %) found the use of supplements necessary during this period. The most commonly used dietary supplements were fish oil (81·9 %), vitamin D (39·0 %), multivitamin supplements (27·4 %), probiotics (22·3 %) and vitamin C (19·4 %), respectively. Most of the participants (62·9 %) stated that they used these supplements once a day to avoid COVID-19 (*n* 293; 94·5 %). The dietitians who used dietary supplements were asked whether they received any advice for the use of supplements, and it was found that the number of the dietitians who did and that of the dietitians who did not were close. The majority of the dietitians who stated that they received advice (45·1 %) had consulted a doctor (Table 3).

**Table 3:** Use of dietary supplements by dietitians during the pandemic period

	*n*	%
Using the dietary supplements is necessary during the pandemic period		
Yes	301	54·7
No	249	45·3
Using of dietary supplements		
Before pandemic period	233	42·4
During pandemic period	77	14·0
No	240	43·6
Type of dietary supplement*		
Fish oil	254	81·9
Vitamin D	121	39·0
Multivitamin	85	27·4
Probiotics	69	22·3
Vitamin C	60	19·4
Black elderberry	54	17·4
Zn	49	15·8
Fe	32	10·3
Vitamin B_12_	16	5·2
Others (folic acid, Se, Ca, Mg	30	9·6
Betaglucan, propolis, coenzyme Q10)		
Frequency of use		
Once a day	195	62·9
Twice a day	22	7·1
3 a day	5	1·6
Every other day	47	15·2
Once a week	28	9·0
2 times a month	13	4·2
Reason for use*		
Protecting from COVID-19	293	94·5
Body weight control	7	2·3
Treatment of diseases	51	16·5
Feeling tired	61	19·7
Not having adequate and balanced nutrition	32	10·3
Using of the dietary supplements had been advised		
Yes	157	50·6
No	153	49·4
Whom was advised for using of the dietary supplements*		
Doctor	140	45·1
Pharmacist	74	23·9
Literature	68	21·9
Medicine company	28	9·0
Friends	11	3·5
TV-radio programmes	3	1·0

* The total number is greater than *n* due to participants could choose more than one response.

While the rate of using functional foods during the pandemic period was 86·0 %, the rate of using herbal medicines was 44·5 %. The findings showed that the dietitians used functional foods to maintain intestinal health (38·5 %), lead a healthy life (36·8 %), avoid COVID-19 (34·9 %), herbal medicines to avoid COVID-19 (46·1 %) and lead a healthy life (41·6 %) (Table 4).

**Table 4:** Using of functional foods and herbal medicines by dietitians during the pandemic period

	*n*	%
Using of functional foods is necessary during the pandemic period		
Yes	503	91·5
No	47	8·5
Using of functional foods		
Yes	473	86·0
No	77	14·0
Reason for use*		
For good health	174	36·8
Body weight control	38	8·0
Mental health	52	11·3
Bowel health	182	38·5
Prevention of diseases	96	20·3
Protect against COVID-19	165	34·9
Using herbal medicine is necessary during pandemic period		
Yes	276	50·2
No	274	49·8
Using of herbal medicine		
Yes	245	44·5
No	305	55·5
Reason for use*		
For good health	102	41·6
Body weight control	35	14·3
Mental health	15	6·1
Bowel health	28	11·4
Prevent diseases	66	26·9
Protect against COVID-19	113	46·1

* The total number is greater than n due to participants could choose more than one response

**Fig. 1:**
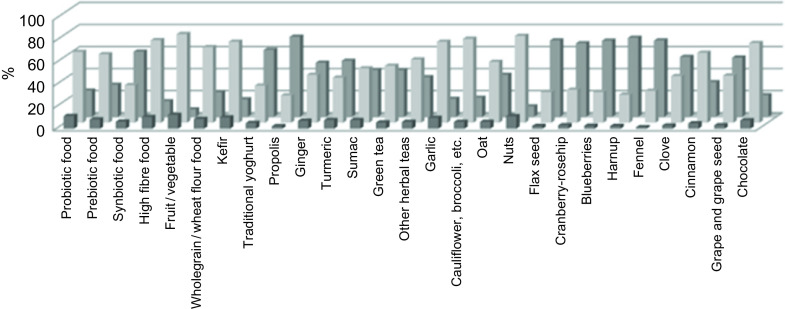
Use of functional foods and herbal medicines. 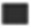, after pandemic; 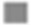, before pandemic; 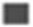, never consumption

In the current study, the use of fifty-seven functional foods and herbal medicines was investigated. The most commonly used ones are summarised in Fig. 1. Foods with functional bioactive compounds, such as foods containing probiotics (64·4 %) and prebiotics (62·2 %), high-fibre foods (41·2 %), vegetables-fruits (80·5 %), whole grain foods (68·7 %), kefir (73·6 %), green tea (51·8 %), other herbal teas (57·5 %), garlic (73·5 %), cauliflower–broccoli–cabbage (76·2 %), nuts (78·9 %) and chocolate (72·4 %), which have beneficial health effects, were already used by the dietitians before the pandemic. Most of the herbal medicines, on the other hand, were used in lower amounts by the dietitians. It was observed that the most commonly used herbal medicines before the pandemic were sumac (49·5 %) and cinnamon (63·5 %). During the pandemic, the dietitians’ use of vegetables/fruits, probiotic foods, nuts, high fibre foods, kefir and garlic increased, respectively.

In Table 5, individuals’ use of functional foods and herbal medicines during the pandemic was evaluated based on gender, age, career year and BMI classification. The use of functional products during the pandemic did not depend on gender and age groups (*P* > 0·05). It was seen that more than half of the dietitians (53·3 %) who used functional foods were at the 1–5th year in their career, 13·3 % of them in their 11–15th year and 13·1 % of them in their ≥ 16th year, whereas 6·5 % of the dietitians who did not use functional foods were in their 11–15th year and 22·1 % of them in their ≥ 16th year (*P* > 0·05). Based on the BMI values, while the rates of using functional foods by dietitians with normal body weight were similar, 3 % of the obese individuals used functional foods during the pandemic and 15·9 % did not (*P* < 0·001). When the herbal medicine use of the participants during the pandemic was evaluated, no statistical difference was observed depending on gender but age, career year and BMI. It was found that approximately three-quarters (73·5 %) of dietitians who used herbal medicines during the pandemic and 63 % of the dietitians who did not were between the ages of 21 and 31 years (*P* < 0·05). Similarly, it was seen that more than half of the individuals (63·3 %) who used herbal medicines and 46·9 % of the dietitians who did not were in their 1–5th year in their career (*P* < 0·05). It was observed that 9·0 % of the participating dietitians who used herbal medicines during the pandemic were overweight and 3·3 % were obese, while 15·4 % of the dietitians who did not use herbal medicines were overweight and 5·9 % were obese (*P* < 0·05).

**Table 5:** Use of functional food and herbal medicines according to gender, age group, professional year and obesity status of dietitians during the pandemic period

	Using of functional foods		Not using of functional foods		Using of herbal medicines		Not using of herbal medicines	
	*n* (473)	%	*n* (77)	%	*n* (245)	%	*n* (305)	%
Gender								
Female	423	89·4	62	80·5	216	88·2	269	88·2
Male	50	10·6	15	19·5	29	11·8	36	11·8
	*P* = 0·350				*P* = 0·540			
Age (years)								
21–31	316	66·8	56	72·7	180	73·5	192	63·0
32–42	114	24·1	10	13·0	48	19·6	76	24·9
43–53	26	5·5	7	9·1	12	4·9	21	6·9
≥ 54	17	3·6	4	5·2	5	2·0	16	5·2
	*P* = 0·083				*P* = 0·036*			
Work experience (years)								
1–5	252	53·3	46	59·7	155	63·3	143	46·9
6–10	96	20·3	9	11·7	43	17·6	62	20·3
11–15	63	13·3	5	6·5	28	11·4	40	13·1
≥ 16	62	13·1	17	22·1	19	7·8	60	19·7
	*P* = 0·250				*P* = 0·036*			
BMI								
Underweight	46	9·7	6	7·8	23	9·4	29	9·5
Normal	353	74·6	50	64·9	192	78·4	211	69·2
Overweight	60	12·7	9	11·7	22	9·0	47	15·4
Obese	14	3·0	12	15·6	8	3·3	18	5·9
	*P* < 0·001*				*P* = 0·044*			

* Chi square test, *P* < 0·05.

The findings obtained in the current study showed that the use of functional foods by women was 2·089 times higher compared with men (95 % Cl: 1·048, 4·165; *P* < 0·05). The use of both functional foods and herbal medicines by dietitians increased with the number of years they spent in their careers. While the use of functional foods increased by 0·063 times in the dietitians with a career of 11–15 years, the use of herbal medicines increased by 0·05 times in dietitians with a career of ≥ 16 years (*P* < 0·05). In addition, the higher the BMI was, the higher the dietitians’ use of functional foods was (*P* < 0·05) (Table 6).

**Table 6:** Evaluation of the effect of gender, age, professional year, obesity and diseases on using of functional foods and herbal medicines use in dietitians by logistic regression

Variables	Using of Functional Foods				Using of Herbal Medicines			
	OR	95 % CI		*P* value	OR	95 % CI		*P* value
Gender								
Male	1				1			
Female	2·089	1·048	4·165	0·036*	0·926	0·528	1·624	0·788
Age (years)								
21–31	1				1			
32–42	7·608	0·687	84·314	0·980	4·858	0·853	27·660	0·075
43–53	0·801	0·174	3·690	0·776	2·471	0·510	11·964	0·261
≥ 54	0·915	0·218	3·846	0·904	0·532	0·152	1·854	0·322
Having chronic disease								
Yes	1				1			
No	1·324	0·706	0·2482	0·382	0·785	0·500	1·232	0·293
Work experience (years)								
1–5	1				1			
6–10	0·107	0·013	0·911	0·041*	0·062	0·015	0·258	0·000*
11–15	0·063	0·007	0·545	0·012*	0·111	0·028	0·440	0·002*
≥ 16	0·392	0·096	1·590	0·190	0·183	0·050	0·674	0·011*
BMI								
Underweight	1				1			
Normal	0·182	0·053	0·627	0·007*	0·742	0·256	2·155	0·584
Overweight	0·180	0·072	0·447	0·000*	0·616	0·247	1·535	0·298
Obese	0·183	0·060	0·551	0·003*	1·210	0·436	3·362	0·714

* Logistic regression, *P* < 0·05.

## Discussion

In recent years, as complementary and alternative therapies have become popular in the world, interest in dietary supplements, functional foods and herbal medicines has started to climb. The idea that they can be effective in the prevention and treatment of the disease during the extraordinary situation we experience today, the pandemic, has caused this interest to increase even more. This growing interest is seen in all age groups, from children to adults and the elderly. Dietitians have undertaken a critical role in directing it individually and socially. Dietitians can both consume functional foods, dietary supplements and herbal medicines and recommend them to their clients/patients, in line with the education they have received and in the light of the evidence-based information^(16,17)^.

Although there are studies in the literature about dietitians’ attitudes towards, knowledge about, and use of dietary supplements, functional foods and herbal medicines, to our knowledge, no study has been carried out on the attitudes of dietitians on this issue and their use of these products during a pandemic. The current study is the first to investigate the attitudes and behaviours of the dietitians in Turkey, regarding how they perceive and use dietary supplements, functional foods and herbal medicines during the COVID-19 pandemic.

When the change in eating habits is questioned, it was seen that the number of dieticians whose eating habits changed positively during the pandemic and that of those whose eating habits remained the same are close (35·1 and 35·3 %, respectively). Similar to our study, the majority of the participants (49·6 %) in the study conducted by Scarmozzino and Visioli in Italy during the pandemic stated that they did not have any significant changes in their diet^(18)^.

In a study conducted on the use of functional foods by dietitians outside the pandemic, the findings showed that more than half (58 %) of 100 dietitians randomly selected used functional foods, and vegetables and fruits were the most commonly used functional foods. Among the dietitians participating in the current study, the ages of the dietitians who used functional foods most frequently ranged between 46 and 55 (63 %), and they were in the 6–15th year of their career. In total, 84 % of the dietitians using functional foods stated that they could be used to prevent diseases and protect health^(19)^. In another study conducted on 385 dietitians in the USA, 74·9 % of the dietitians indicated that the use of functional foods was effective in providing protection against diseases and improving health^(20)^. In the study conducted by De Jong *et al.* on Dutch dietitians, they found out that 69 % of the dietitians consumed little or no functional food. Functional foods consumed most commonly by the dietitians were foods enriched with vitamins (*n* 38), probiotics (*n* 34) and margarine enriched with phytosterols (*n* 16)^(21)^. Unlike the studies referred to, the current study found that the dietitians used more functional foods (86·0 %), which was due to being conducted during the pandemic. It was also stated that these foods were used to maintain intestinal health (38·5 %), lead a healthy life (36·8 %) and avoid COVID-19 (34·9 %). Consistent with other studies, the current study found that functional foods most commonly used both during and before the pandemic were vegetables and fruits (80·5 %), nuts (78·9 %), kefir (73·6 %) and garlic (73·5 %), depending on the dietary habits in our country.

Dietitians can evaluate the adequacy of nutrient intake and advice on the use of dietary supplements in cases of any deficiencies. The tendency towards the use of dietary supplements compared with functional foods increases even more during pandemics to boost the immune system and to eliminate nutrient deficiencies. Many studies have been conducted in different countries to evaluate the use of dietary supplements by dietitians and their attitudes^(22–26)^. In a study conducted in California, USA, it was observed that the rate of dietary supplement use by dietitians was 69 %, and the most commonly used dietary supplements were Ca, multivitamins, vitamins E and C and Fe, respectively. The most common underlying reasons for using dietary supplements included ensuring adequate intake, preventing osteoporosis, facilitating wound healing, consuming antioxidants, preventing anaemia, having a healthy pregnancy and improving overall health^(22)^. A study conducted in the USA found that the prevalence of regularly using dietary supplements by dietitians was 74 % and 84 % of the dietitians using dietary supplements used multivitamins, 63 % used Ca, 47 % used fish oil and 43 % used vitamin D. Another study found that 68 % of the dietitians used multivitamins. The majority (43 %) used them every day as in the current study^(23,24)^. The reasons for using dietary supplements were found to be maintaining bone health (58 %), improving overall health, leading a healthy life (53 %) and eliminating nutrient deficiencies (42 %) due to malnutrition^(23)^. Similarly, a study conducted by Lederman *et al.* on healthcare professionals in the USA found that 66 % of the dietitians used dietary supplements to protect their health, 42 % to treat diseases and 19 % to enhance physical and mental performance^(25)^. The small-sample (*n* 95) study conducted on dietitians in the Netherlands found that 38 % of the participants frequently used dietary supplements to protect the health, 22 % to treat diseases and 13 % to enhance physical and mental performance. It was determined that 84 % of the dietitians used vitamin supplements and 60 % mineral supplements in the last 5 years. The most commonly used vitamins were multivitamins, vitamins D, C, and B complex and the most commonly used minerals were Ca, Fe and Mg^(26)^. In a study conducted on 367 South African dietitians, 51 % of them stated that they used multivitamins and minerals at least three times a week, and 35 % stated that they used them every day. It was found that Ca (17·8 %) and fish oil (17·3 %) were commonly used as daily dietary supplements^(27)^. Compared with the studies conducted in many countries, the current study found that the use of dietary supplements by dietitians was lower (56·4 %), despite the pandemic, that only 54·7 % of them found it necessary to use dietary supplements during this period, and most of them (% 62) only used these supplements once a day. Similar to the studies conducted, the most commonly used dietary supplements in the current study were fish oil (81·9 %), vitamin D (39·0 %), multivitamin (27·4 %), probiotics (22·3 %) and vitamin C (19·4 %). It was seen that the majority of the dietitians used dietary supplements to avoid COVID-19 (*n* 293; 94·5 %). When compared with the results of the studies conducted in other countries, the use of dietary supplements was lower in the current study despite the pandemic. One of the reasons for this was that dietitians, who had an academic background in nutrition, were aware that dietary supplements would not be needed as long as an adequate and balanced diet was ensured. However, awareness may not always be enough. A study published in Italy during the pandemic suggested that the Mediterranean diet is no longer adequate in high energy and micronutrients as the Western diet, due to the globalisation of food production and supply systems in Spain and Italy, which are part of the Mediterranean Diet^(28)^. Although our country is also a Mediterranean country, this does not apply to Turkey. When the nutritional habits of our country are reviewed, it is seen that 48 % of the population consume green-leafy vegetables, 34·9 % of them consume other vegetables and 52 % of them consume fruits every day^(29,30)^ (TÜBER, 2015; TBSA, 2019). We can easily reach fish, nuts, whole grains, vegetables and fruits that contain macro and micronutrients to boost our immune system, and we often prefer them in our daily diet, which reduces the rate of dietary supplement use.

It was observed that the use of herbal medicines by dietitians was lower compared with that of dietary supplements and functional foods^(19,23,25,26,31,32)^. In a study conducted on dietitians in the USA, only 19 % of dietitians stated that they used herbal medicines. It was observed that dietitians, who were between the ages of 26 and 35 years (50 %) and in the 6–15th year of their career (63 %), used herbal medicines most commonly^(19)^. In the study conducted by Hetherwick *et al.* on 253 dietitians in California, the findings showed that 18 % of the dietitians used herbal medicines, and the most commonly used one was Echinacea. The reasons for using herbal medicines included enhancing the immune system, treating menopause, improving memory and prostate health^(22)^. In a small-sample (*n* 89) study conducted in Florida, the findings showed that 28 % of dietitians used herbal medicines to protect the health, 32 % to treat diseases and 16 % to increase physical and mental performance^(25)^. Another study found that less than half (42 %) of dietitians used herbal medicines^(31)^. Similarly, in the study conducted by Cashman *et al.* on 158 dietitians, the findings showed that 37 % of dietitians used herbal medicines. The most commonly used product was Echinacea (80 %), and there was an inverse correlation between the use of herbal medicines and the career year (*r* = –0·45, *P* = 0·012)^(32)^. In the study conducted on Dutch dietitians, it was observed that the personal use of herbal medicines was significantly lower (18 %), and green tea was one of the most commonly used ones^(26)^. Consistent with the studies in the literature, the current study also found that the rate of using herbal medicines by dietitians during this period was 44·5 %, despite the pandemic, and the reasons for using them included avoiding COVID-19 (46·1 %) and leading a healthy life (41·6 %). It was observed that the rate of use increased in dietitians who were young (73·5 % were at the ages of 21–31 years) and who were in the early years of their career (63·3 % of them were at the 1–5th years of their career).

Our study had some limitations to consider. First, the study data were collected by means of a self-reported web-based questionnaire due to the COVID-19 pandemic restrictions. However, our web-based questionnaire was similar to others that have been frequently employed. Second, the current study included dietitians in Turkey; therefore, the results might not be applicable to the rest of the world. In the future, similar and more extensive studies should be conducted on dietitians worldwide, which can will help the public patients prepare their response to the unavoidable pandemics in the future.

## Conclusion

During the pandemic, the tendency to use dietary supplements instead of natural foods increases. More emphasis is placed on the use of dietary foods and herbal medicines with nutrients and functional bioactive compounds that are effective in boosting immunity, have protective effects against diseases and do not cause any negative effects. As in the COVID-19 pandemic, in cases where the immune system gains significance in the protection from diseases, attention should be paid to adequate and balanced nutrition, and daily intake of macro and micronutrients should be met based on age and gender. Individuals who do not have an adequate and balanced diet should start using dietary supplements, functional foods and herbal medicines under the control of a physician and dietitians after their general dietary habits are evaluated. Since dietitians could best evaluate their general dietary habits, they are more controlled in using the specified foods and products.

## References

[1] Republic of Turkey Ministry of Health (2020) What is COVID-19 (New Coronavirus Disease)? https://covid19bilgi.saglik.gov.tr/tr/covid-19-yeni-koronavirus-hastaligi-nedir.html (accessed June 2020).

[2] Tosh PK (2020) Coronavirus: what is it and how can I protect myself? https://www.mayoclinic.org/diseases-conditions/coronavirus/expert-answers/novel-coronavirus/faq-20478727 (accessed June 2020).

[3] Unicef (2020) Coronavirus disease 2019 (COVID-19): What is it really? https://www.unicef.org/wca/what-is-coronavirus (accessed June 2020).

[4] World Health Organization (2020) Coronavirus Disease (COVID-19) Dashboard. https://www.who.int (accessed September 2020).

[5] World Health Organization (2020) Coronavirus. https://www.who.int/health-topics/coronavirus#tab=tab_1 (accessed June 2020).

[6] Turkish Dietetic Association (2020) COVID-19 Nutrition Recommendations. http://www.tdd.org.tr/index.php/duyurular/69-covid-19-beslenme-onerileri (accessed June 2020).

[7] Acar Tek N & Pekcan G (2008) Should Food Supplements Be Used? Ankara: Klasmat Printing.

[8] Ashwell M (2002) Concepts of Functional Foods. ILSI Europe Concise Monograph Series. https://ilsi.eu/wp-content/uploads/sites/3/2016/06/C2002Con_Food.pdf (accessed June 2020).

[9] Alkhatib A , Tsang C , Tiss A et al. (2017) Functional foods and lifestyle approaches for diabetes prevention and management. Nutrients 9, 1310. 10.3390/nu9121310 PMC5748760 29194424

[10] del Castillo MD , Iriondo-DeDond A & Martirosyan DM (2018) Are functional foods essential for sustainable health? Ann Nutr Food Sci 2, 1015.

[11] European Commission (2010) Functional Foods. Luxembourg: Publications Office of the European Union.

[12] Markowiak P & Slizewska K (2017) Effects of Probiotics, Prebiotics, and Synbiotics on human health. Nutrients 9, 1021. 10.3390/nu9091021 PMC5622781 28914794

[13] Turkish Medical Association (2012) Herbal Products and Health: Scientific and Ethical Approach, pp. 10–29. Ankara: Turkish Medical Association Publication. https://www.ttb.org.tr/kutuphane/bitkisel.pdf (accessed October 2020).

[14] Ozdemir B , Sahin I , Kapucu H et al. (2013) How safe is the use of herbal weight-loss products sold over the Internet? Hum Exp Toxicol 32, 101–106. 22354083 10.1177/0960327112436407

[15] Ohnishi N & Yokoyama T (2004) Interactions between medicines and functional foods or dietary supplements. Keio J Med 53, 137–150. 15477727 10.2302/kjm.53.137

[16] Radimer K , Bindewald B , Hughes J et al. (2004). Dietary supplement use by US adults: data from the National Health and Nutrition Examination Survey, 1999–2000. Am J Epidemiol 160, 339–349. 15286019 10.1093/aje/kwh207

[17] Hathcock J (2001) Dietary supplements: how they are used and regulated. J Nutr 131, S1114–S1117. 10.1093/jn/131.3.1114S 11238828

[18] Scarmozzino F & Visioli F (2020) Covid-19 and the subsequent lockdown modified dietary habits of almost half the population in an Italian sample. Foods 9, 675. 10.3390/foods9050675 PMC7278864 32466106

[19] Monahan Couch L , Harris JE (2008) Knowledge, attitudes, and self-reported practices of pennsylvania registered dietitians regarding functional foods and herbal medicine. Top Clin Nutr 23, 32–46.

[20] Berhaupt-Glickstein A & Enrione EB (2011) Functional foods perceptions, attitudes, and practices of registered dietitians. Top Clin Nutr 26, 312–323.

[21] de Jong N , Hoendervangers CT , Bleeker JK et al. (2004) The opinion of Dutch dietitians about functional foods. J Hum Nutr Diet 17, 55–62. 14718032 10.1046/j.1365-277x.2003.00498.x

[22] Hetherwick C , Morris MN & Silliman K (2006) Perceived knowledge, attitudes, and practices of California registered dietitians regarding dietary supplements. J Am Diet Assoc 106, 438–442. 16503236 10.1016/j.jada.2005.12.005

[23] Dickinson A , Bonci L , Boyon N et al. (2012) Dietitians use and recommend dietary supplements: report of a survey. Nutr J 11, 14. 22416673 10.1186/1475-2891-11-14 PMC3331817

[24] White JV , Pitman S & Blumberg JB (2007) Dietitians and multivitamin use: personal and professional practices. Nutr Today 42, 62–68.

[25] Lederman VG , Huffman FG & Enrione EB (2009) Practices, attitudes, and beliefs regarding dietary supplements among Florida’s dietitians and nurses. J Diet Suppl 6, 124–142. 22435413 10.1080/19390210902861833

[26] Ten Hoeve AL (2011) Dietitians in the Netherlands and dietary supplements: practices, personal use and beliefs. Int J Nutr Metab 3, 11–16.

[27] Steyn NP , Labadarios D , Nel JH et al. (2005) Development and validation of a questionnaire to test knowledge and, practices of dietitians regarding dietary supplements. Nutrition 21, 51–58. 15661478 10.1016/j.nut.2004.09.008

[28] Galli F , Reglero G , Bartolini D et al. (2020) Better prepare for the next one. Lifestyle lessons from the COVID-19 pandemic. Pharma Nutr 12, 100193. 10.1016/j.phanu.2020.100193 PMC7202804 32382499

[29] Turkey Dietary Guidelines 2015 (TÜBER) (2016) Republic of Turkey Ministry of Health Publication Number: 1031. Ankara. https://dosyasb.saglik.gov.tr/Eklenti/10915,tuber-turkiye-beslenme-rehberipdf.pdf (accessed December 2020).

[30] Turkey Nutrition and Health Survey (TBSA) (2019) Republic of Turkey Ministry of Health Publication Number: 1132. Ankara. https://hsgm.saglik.gov.tr/depo/birimler/saglikli-beslenme-hareketli-hayat-db/Yayinlar/kitaplar/TBSA_RAPOR_KITAP_20.08.pdf (accessed December 2020).

[31] Lee YK , Georgiou C & Raab C (2000) The knowledge, attitudes, and practices of dietitians licensed in Oregon regarding functional foods, nutrient supplements, and herbs as complementary medicine. J Am Diet Assoc 100, 543–548. 10812379 10.1016/S0002-8223(00)00169-3

[32] Cashman LS , Burns JT , Otieno IM et al. (2003) Massachusetts registered dietitians’ knowledge, attitudes, opinions, personal use, and recommendations to clients about herbal supplements. J Altern Complem Med 9, 735–746. 10.1089/107555303322524580 14629851

